# Requirements for (web-based) physical activity interventions targeting adults above the age of 65 years – qualitative results regarding acceptance and needs of participants and non-participants

**DOI:** 10.1186/s12889-020-08927-8

**Published:** 2020-06-11

**Authors:** Frauke Wichmann, Claudia R. Pischke, Dorothee Jürgens, Ingrid Darmann-Finck, Frauke Koppelin, Sonia Lippke, Alexander Pauls, Manuela Peters, Claudia Voelcker-Rehage, Saskia Muellmann

**Affiliations:** 1 Institute for Public Health und Nursing Sciences – IPP, University of Bremen, Bremen, Germany; 2 Department Prevention and Evaluation, Leibniz Institute for Prevention Research and Epidemiology – BIPS, Bremen, Germany; 3 Institute of Medical Sociology, Centre for Health and Society, Medical Faculty, Heinrich-Heine-University Duesseldorf, Duesseldorf, Germany; 4 Jade University of Applied Sciences Wilhelmshaven/Oldenburg/Elsfleth, Section Technology and Health for Humans, Oldenburg, Germany; 5 Department of Psychology & Methods, Jacobs University Bremen, Bremen, Germany; 6 Research Focus Health Sciences Bremen, University of Bremen, Bremen, Germany; 7 Institute of Human Movement Science and Health, Faculty of Behavioural and Social Sciences, Chemnitz University of Technology, Chemnitz, Germany; 8 Neuromotor Behavior and Exercise, Institute of Sport and Exercise Sciences, University of Muenster, Muenster, Germany

**Keywords:** Physical activity, Older adults, Requirements, Acceptance, Implementation, Web-based, Prevention, Social-ecological model

## Abstract

**Background:**

It remains unclear how physical activity (PA) interventions need to be designed to reach older adults and to be widely accepted in this target group. The aim of this study was to assess the acceptance of a web-based PA program, including individual intervention components as well as relevant contextual factors, and to specify requirements for future interventions.

**Methods:**

Two hundred sixty-six participants of a PA intervention completed a questionnaire covering individual program components (content, structure, and context). Further, 25 episodic guided interviews focusing on reasons for (non-) participation were conducted with 8 participants and 17 non-participants. Following qualitative content analysis, different requirements were identified and organized based on the social-ecological model, resulting in a profile of requirements.

**Results:**

Based on the participants’ and non-participants’ statements, six different levels of requirements affecting acceptance of and successful participation in a web-based PA intervention were identified. The individual fit was influenced by an interaction of different factors at the intrapersonal, sociocultural, content, spatial, digital and organizational levels. Several age- and gender-specific requirements were noted in the interviewed older adults. Men and women, as well as younger (< 70 years) and older (≥70 years) adults differed in terms of perceived enjoyment and benefits of socializing while exercising together, the time expenditure perceived to be acceptable, previous digital skills, as well as in perceptions that ambience and accessibility of exercise facilities in the neighborhood were important.

**Conclusions:**

To motivate older adults to engage in PA and address different needs in terms of life circumstances and quality of life as well as differences in technical affinity, different requirement profiles should be included in the process of intervention development and implementation. Participatory development loops and modular offer formats are recommended for this.

## Background

Physical inactivity is a global problem contributing to the etiology of chronic diseases [1], such as cardiovascular diseases or type II diabetes in older age [2, 3]. For this reason, the World Health Organisation (WHO) and the American College of Sports Medicine (ACSM) recommend that adults above the age of 65 years engage in at least 150 min per week of moderate-to-vigorous physical activity (PA) and strengthening exercises twice per week [4, 5]. In Germany, approximately one fifth of individuals above this age (24% of men, 17% of women) reach these recommendations [6]. As in other industrialized countries, the demography in Germany is changing. The percentage of older adults in the population and life expectancy is increasing, leading to a need for new health policies and concepts for health promotion. Due to the increased use and acceptance of the internet and mobile end devices in older adults [7, 8], web-based interventions for PA promotion are increasingly adopted in health promotion. These have been shown to be effective over the short-term [9, 10].

The success of these interventions becomes tangible when PA interventions fit with individual goals, expectations, and motives to engage in PA, previous experiences, as well as health and life conditions [11]. This however requires the close consideration and understanding of the target group. Initiators of PA interventions are frequently confronted with a high level of heterogeneity of the older population [12, 13]. For example, there are differences in participation in health promotion programs (including interventions to promote PA) by social class and gender [14]. Regarding exercise behavior, a representative study of older adults in Germany found differences in factors facilitating or impeding exercise by age group (≤80 vs. > 80 years), gender and living situation (e.g., number of persons in the household) [15, 16]. Similarly, in a qualitative study involving 33 participants that focused on experiences with the use of health technologies for PA, mainly female respondents were observed to be more interested in the use of digital technologies for PA [17].

Good physical and mental health are important preconditions for participating in PA among older adults [13, 18, 19], hence impaired health is often a major barrier [20, 21]. Further, positive experiences with PA in the past and generally enjoying being physically active contribute towards a physically active lifestyle [21, 22]. PA is also often regarded as a way to get or remain in touch with other people [18]. The personal contact between participants and coaches also increases the likelihood that older adults will remain physically active [23]. Spatial and structural conditions (e.g., perceived attractiveness and access of the location of the intervention) [22, 24, 25] as well as equipment [22] are further relevant factors for participation in PA programs. Another factor influencing PA is the time of the day the intervention is offered [24]. Activities offered during the evenings are generally perceived as being less attractive as this means that participants will go back home in the dark, especially during wintertime.

Research examining optimal characteristics of PA interventions targeting older adults suggests that the PA done should be of low to medium intensity [23], but also that challenging PA is not necessarily a barrier to participation [21]. Web-based interventions should be kept simple [26] and allow for goal setting [27]. To date, interventions that have been shown to be effective comprise multiple intervention components, such as individualized/tailored PA recommendations, monitoring of PA behavior (e.g., via digital PA diaries), and tailored feedback [9]. In addition, the teams developing these interventions ensured that the information provided met the participants’ expectations, needs, and abilities [28].

To further the understanding of the dynamics involved in the interaction between individuals (e.g., older adults) and the environment (e.g., single factors influencing PA), social-ecological models or frameworks have proved to be useful [29]. In these models, psychosocial factors impacting on behavior change, for instance previous experiences, knowledge, attitudes, expectations, and motivation as well as contextual factors such as factors at the organisational, ecological, and political levels, are integrated. Boulton et al. [30] developed a social-ecological model of PA behavior change based on results of focus groups and semi-structured interviews conducted with 60 individuals aged 50 years and above, and identified the following five levels of the social-ecological model that influenced PA engagement: individual, intrapersonal, perceived environment, organisational and political. According to the authors, these levels are relevant with regard to PA among older adults. Specific requirements for the design and implementation of web-based PA interventions targeting older adults in Germany have not yet been assessed. The aims of this qualitative research were to (i) identify requirements of older adults regarding participation in web-based interventions for PA promotion and (ii) derive differentiated profiles summarizing the requirements and informing the development and implementation of future PA interventions for the target group of individuals aged 65 years and above.

## Materials and methods

### Data assessment

The starting point of this qualitative research were the two studies PROMOTE [31] and Ready to Change (RTC) [32], which were both subprojects of the prevention research network Physical activity and health equity: primary prevention for healthy ageing (AEQUIPA) [33] funded by the German Federal Ministry of Education and Research (BMBF).

In the PROMOTE study, the effectiveness of participating in two ten-week web-based PA interventions was assessed in adults aged between 65 and 75 years and compared to a waitlisted control group [31, 34, 35]. The two web-based interventions were developed theory-based according to self-regulation theory [36] and principles of behavioral change [37]. Participants of the two intervention groups were enrolled in the ‚Fit in the Northwest‘ program and monitored their PA subjectively using a web-based PA diary (intervention group 1), or subjectively *and* objectively, using the web-based PA diary and a Fitbit Zip to monitor steps per day (intervention group 2). Based on the individual activity level for baseline and gender, participants from the two intervention groups received an exercise catalogue with age-appropriate exercises according to the PA recommendations of the WHO. The exercise catalogues also contained a list of sports activities offered in the respective communities. In addition, weekly group meetings were organized for the participants, during which technical problems were addressed and some theoretical background regarding PA promotion (e.g., relevance of social support for PA promotion) was provided. The participants could also exercise in groups (walks, strength and balance training). The waitlisted control group participants received access to the intervention of intervention group 1 after the completion of the study. Both intervention group participants received a questionnaire assessing acceptance of the intervention at 12-weeks follow-up [31, 34]. The data assessment and implementation of the intervention took place between May 2016 and November 2017. All participants provided written consent to participate in the study.

In RTC, qualitative episodic guided interviews were conducted with participants and non-participants of the PROMOTE study to assess the reasons for participation, respectively, non-participation in PA interventions [38]. The sample for this qualitative study was created after the recruitment for the PROMOTE study had begun. The target group included adults aged 65 to 75 years who had either decided to participate in the study (participants) or not to participate (non-participants). Participants and non-participants were recruited at a ratio of 2:1 using two strategies: direct recruitment (e.g., via flyers and written contact) and indirect strategies (e.g., snowball system). The interviews were conducted between March and May 2017. All interviews were recorded, transcribed verbatim, and analyzed using the software MAXQDA (version 10). Further detail regarding the recruitment of interviewees is reported elsewhere [38].

### Data analysis

This paper is based on the results of the questionnaire-based survey assessing acceptance of the intervention among the two intervention group participants of PROMOTE, that was conducted at the 12-weeks follow-up, as well as the qualitative semi-structured interviews which were conducted as part of RTC (see Table 1 for further details regarding the content of the survey questionnaire and the interview guide). Due to the heterogeneity of the data material, qualitative content analysis was chosen to analyse the data [39]. Following a deductive-inductive approach, a theoretical frame containing key topics and subtopics was developed. The classification of the relevant text and coding units to the overarching categories as well as the subcategories was performed by two independent researchers. The developed framework was systematically applied to the entire dataset and finalized following an iterative process (discussion <− > modification). In order to reduce the risk of bias, peer debriefing strategies were implemented. The results of individual analysis steps were elaborated and further developed by two scientists at regular intervals (four eyes principle). Important milestones of data analysis (overarching categories and socio-ecological model) were validated in two workshops attended by three qualitatively researching scientists. For instance, selected codes reflected upon and discussed together. All deviations in interpretation were discussed until a consensus was reached. Illustrative quotes were selected from the existing material as anchor examples. In addition, differences in statements between younger (< 70 years) and older (≥70 years) participants, males and females as well as those with low, medium and high levels of education were analyzed. Following the 2011 version of the International Standard of Education (ISCED) [40], level of education was categorized as low (ISCED score 1–2), medium (ISCED score 3–4), or high (ISCED score 5–8). Excerpts of the interviews and questionnaires relevant for this manuscript were translated from German into English (see Tables 2 and 3). To minimize the risk of possible blurring and translation errors, all English quotes were checked against original transcripts by a team consisting of the interviewer and a native speaker.

**Table 1:** PA-related questions for the subprojects PROMOTE and RTC

**Questionnaire**^**a**^**:**	
Why would you recommend the ‚Fit in the Northwest‘ program?	
Why would you not recommend the ‚Fit in the Northwest‘ program?	
What was missing in the ‚Fit in the Northwest‘ program?	
Which recommendations for improving the program do you have?	
**Interview guide**^**b**^**:**	
Which parts of the PA intervention appeal to you?	
Which parts of the intervention did you not like?	
How does participating in the intervention benefit you personally?^c^	
How do you rate the location, access, costs, scheduling and degree of familiarity/popularity?^c^	
How do you rate the provider and the relatedness with the community of the intervention?	
From your point of view, which individuals participate in the intervention?	
What do you think about the persons offering the intervention?	

a: PROMOTE; b: RTC; c: Only participants of the interventions were asked this question; PA: physical activity

**Table 2:** Characteristics of respondents from the PROMOTE and RTC studies

Charactersitsics	PROMOTE (***n*** = 266)	RTC (***n*** = 25)	Total (***n*** = 291)
**Age, n (%)**			
< 70 years	143 (53.7)	10 (40.0)	153 (52.6)
≥ 70 years	113 (42.5)	15 (60.0)	128 (44.0)
Missing	10 (3.7)		10 (3.4)
**Gender, n (%)**			
Men	117 (44.0)	10 (40.0)	127 (43.6)
Women	146 (55.0)	15 (60.0)	161 (55.3)
Missing	3 (1.0)		3 (1.0)
**Community, n (%)**			
Obervieland	84 (31.6)		84 (28.9)
Vahr	25 (9.4)	13 (52.0)	38 (13.1)
Burglesum	35 (13.2)	10 (40.0)	45 (15.4)
Achim	43 (16.2)		43 (14.8)
Osterholz-Scharmbeck	79 (29.7)	2 (8.0)	81 (27.8)
**Country of birth, n (%)**			
Germany	256 (96.2)	23 (92.0)	279 (95.9)
Other country	7 (2.6)	2 (8.0)	9 (3.1)
Missing	3 (1.1)		3 (1.0)
**Family status, n (%)**			
Married, living together with spouse	198 (74.4)	11 (44.0)	209 (71.8)
Married, separated from spouse or divorced	28 (10.5)	6 (24.0)	34 (11.7)
Unmarried	8 (3.0)	2 (8.0)	10 (3.4)
Widowed	28 (10.5)	6 (24.0)	34 (11.7)
Missing	4 (1.5)		4 (1.4)
**ISCED, n (%)**			
Low	2 (0.8)		2 (0.7)
Medium	121 (45.5)	19 (76.0)	140 (48.1)
High	134 (50.4)	6 (24.0)	140 (48.1)
Missing	9 (3.4)		9 (3.1)
**WHO MVPA recommendations achieved, n (%)**			
No	29 (10.9)	–	
Yes	235 (88.3)	–	
Missing	2 (0.8)	–	
**MVPA, n (%)**			
Never	–	–	
Once a month	–	–	
Once a week	–	2 (8.0)	
2–3 times a week	–	7 (28.0)	
4 times a week or more often	–	10 (40.0)	
Daily	–	6 (24.0)	
**Intervention group PROMOTE, n (%)**			
Intervention group 1	146 (54.9)	–	
Intervention group 2	120 (45.1)	–	
**Participants/Non-Participants RTC, n (%)**			
Participants	–	8 (32.0)	
Non-partipants	–	17 (68.0)	

*ISCED* International Standard Classification of Education; *MVPA* moderate-to-vigorous PA; *WHO* World Health Organization; (−) no relevant data collected

**Table 3:** Results of the interviews – Examples of quotes^a^

Theme	Number	Quote
**1. Requirements on interpersonal level**		
	Q 1.1	*“Exercising on a regular basis preserves health and well-being. The program is holistic.*”* (participant, female, > 70 years, high level of education)*
	Q 1.2	*“It stimulates you to be physically active. It points out your personal athletic deficits, so you can work on improving them.” (participant, female, > 70 years, high level of education)*
	Q 1.3	*“It motivated me and drove my ambition to do more sports, respectively gymnastics, on a regular basis and the small daily successes supported that.” (participant, female, > 70 years, high level of education)*
	Q 1.4	*“More individualization at the beginning would keep more people on board.” (participant, female, > 70 years, high level of education)*
	Q 1.5	*“More nutrition tips.*”*(participant, female, > 70 years, medium level of education)*
**2. Requirements on intrapersonal or sociocultural level**		
Other participants and social exchange	Q 2.1	*“The group helps me to stick to the program.*” *(participant, female, ≥70 years, medium level of education)*
	Q 2.2	*“Yes, it would be nice if it were a mixed group and not only women would be there.” (participant, female, > 70 years, medium level of education)*
	Q 2.2	*“The groups should not be mixed (…*).*” (participant, female, < 70 years, high level of education)*
	Q 2.3	*“Whereby it is not always good when there are couples in the group, they often argue.” (non-participant, female, ≥70 years, high level of education)*
	Q2.4	*“Yes, I find it good, if my friend with whom I already do a few things would also take part.” (non-participant, female, ≥70 years, medium level of education)*
Trainers/Exercise instructors	Q2.6	*“Well, a professional qualification like the uh, there in, in the fitness field. That people are also trained, not only as fun organizers, but have medical knowledge. That I would like that, yes.” (participant, female, ≥70 years, high level of education)*
	Q 2. 7	*“They were approachable, we could ask them and they were forthcoming. I found that to be very good. They were able to convey it very well and I found that to be very, very good.” (participant, female, > 70 years, medium level of education)*
	Q 2.8	*“Well, then we again come up against the “clubby culture*”*, afterwards if we have someone who goes “it’s this way and not that way and not like that”. I mean, it would have to be a very open. That one doesn’t necessarily say “so, this is our leader and, and …*.” *(non-participant, male, ≥70 years, medium level of education)*
**3. Requirement on the content level of the proposed program (proposed program performed)**		
Exercises, instructions and goal setting	Q 3.1	*“The exercise catalogue, which was divided into different segments, positively surprised me, respectively, enthused me moderately. Good, interesting exercises! Easy to do for everyone and efficient!*“ *(participant, male, high level of education)*
	Q 3.2	*“Perhaps participants should have been separated more according to their level of performance. Particularly for the strength training I would have liked to have other different exercises. The same exercises for ten weeks were monotonous and*.” *(participant, female, > 70 years, medium level of education)*
	Q 3.3	*“I would have liked to have someone to assist me, at least when doing the first exercises, who would check whether the exercises were being done correctly.” (participant, female, < 70 years, medium level of education)*
	Q 3.4	*“Perhaps a CD with the exercises, which one can look at later on and check if the motions are still correct. It can also be for a price.” (participant, male, < 70 years, high level of education)*
	Q 3.5	*“The demands of the program could be raised after four, respectively, eight weeks.” (participant, female, < 70 years, high level of education)*
	Q 3.6	*“Exercises for the mind (memory training).*”* (participant, female, < 70 years, medium level of education)*
Group meetings	Q 3.7	*“Unfortunately, it was like this. In the beginning, when the program started, we were about 20 people, but only four or five showed up for the meetings. These were then also almost always the same people. The one or the other then also came along, but otherwise there were mainly five or six people. Too few. And I really think the exchange is important, so that one can also hear from the others how they go about it (laughs).” (participant, male, ≥70 years, high level of education)*
	Q 3.8	*“It was too much theory and too little exercising together.*” *(participant, female, < 70 years, medium level of education)*
Specific eHealth intervention components	Q 3.9	*“Well, about the logging in regularly and taking notes every day, that was indeed a motivation. The stimulation to do a bit of something each day. I then made sure that I was always at the gym on Monday, Wednesday, and Friday, and on the other two days I tried to cycle a little or to jog once in a while, that is, something that I otherwise had so far avoided.” (participant, female, ≥70 years, high level of education)*
	Q 3.10	*“Entering something into the PC every day was too much.*” *(participant, female, < 70 years, high level of education)*
	Q 3.11	*“Monitoring the number of steps with the Fitbit is informative.*” *(participant, female, ≥70 years, high level of education)*
	Q 3.12	*“The synchronization with the Fitbit-App was at times very tedious (several attempts required).*” *(participant, male, < 70 years, high level of education)*
**4. Requirements on spatial level**		
Accessibility/Reachability	Q 4.1	*“Well, there I would say if it has to be, then close to here in the area, but not too … if I first have to go to the other side of the town,, to the stadium or so in that direction, that is always quite a ride for us. Also not long, but not such that one is already tired from exercising. To the university, that would also be quite fast.” (non-participant, male, < 70 years, medium level of education)*
	Q 4.2	*“And I would even go as far as Osterholz, they are supposed to also have a nice swimming pool.*” *(non-participant, male, < 70 years, medium level of education)*
	Q 4.3	*“When there’s a lot of traffic I would say I am there in 15 to 20 min. That would be possible, yes. One could also use the bike if need be, isn’t it? Well, but I wouldn’t really want to cycle into the city center or wherever else. I wouldn’t have/ I wouldn’t do that. That would be too far for me. That is a quarter of an hour, maximum 20 min.*” *(non-participant, female, > 70 years, medium level of education)*
Location	Q 4.4	*“And the venues, they were not appropriate. Well, I think it is probably difficult to find a suitable location. We sometimes had to do something in a very small area. I mean, it was nobody’s fault, in this, that was in the multigenerational house, sometimes we had the large room, then it was super, there one could move around.” (participant, female, > 70 years, medium level of education)*
	Q 4.5	*“If one does sports, then one can’t only do so in a gym or in such places where there are appropriate (mats?). I think it was okay like that, it was a closed room.” (participant, male, ≥70 years, high level of education)*
	Q 4.6	*“Yes, it should really be a location where there are other things nearby that one needs to do. Let’s say for example if one can do the shopping, other things, that is, everyday stuff things that one can do. So that I can say, okay, “I’ll do a bit of shopping, stop by the post office and then I have to do this and so forth.” That it’s in such an area. Well, and if one also had the chance to be inside or outside, depending on the time of the year, there would be nothing wrong with that, it doesn’t always have to be somewhere outside or only inside, but rather such that one would have different possibilities.” (participant, male, ≥70 years, medium level of education)*
**5. Requirements at the digital level**		
	Q 5.1	*“To design the website better:* i.e. *make it easier for senior participants to understand.*”* (participant, female, ≥70 years, medium level of education)*
	Q 5.2	*“There were participants who had major problems installing the program. Their questions were not, respectively, could not be answered, they gave up, they were somehow excluded and I never saw them again.” (participant, male, > 70 years)*
	Q 5.3	*“(…*) *it would therefore have been better to focus on the title “fit” and to place the sports part at the beginning. The questions and explanations regarding the technique can then be explained at the end if needed.” (participant, male, < 70 years, high level of education)*
**6. Requirements at the organizational level**		
Sequence and duration	Q 6.1	*“Improvement successes, changes in awareness, should be documented and assessed over a longer period.” (participant, male, < 70 years, high level of education)*
	Q 6.2	*“Yes, it was a bit too long I have to say, for me that is. One somehow loses interest a bit afterwards, well, it’s the same thing over and over again. In the end it is only then. At some point one just says*” *Oh well, okay.” (participant, female, < 70 years, medium level of education)*
Scheduling	Q 6.3	*“That would be an obstacle for me and if I could wish for something, that would be some kind of uh, open system, which provides certain things that however do not mean that one is committed to participate at fixed times with certain people. Those for me, would be the ideal conditions.” (non-participant, male, ≥70 years, high level of education)*
Local stakeholders	Q 6.4	*“Representatives from sports club should present their activities during group meetings.*” *(participant, male, < 70 years, high level of education)*
	Q 6.5	*“Of course, there is a financial span. It should not be too expensive, but a certain fee, what one also pays for a sports club membership or wherever, no problem at all.*”*(participant, female, < 70 years, medium level of education)*
**Age-, gender-, and education-related specifics of the requirement profile**		
Age-specific factors	Q A.1	*“Well, the chemistry just has to be right. It somehow has to be people, that one somehow has some common interests.*”*(participant, male, < 70 years, medium level of education)*
	Q A.2	*“Well, in order not to have large differences (laughs), it would be nice if the same age groups would be there, I’d say it should be from 60.” (participant, male, ≥70 years, high level of education)*
	Q A.3	*“But somewhere where one says, okay, they accept you, I accept them. But let’s say like 35 to 40 year olds.*” *(non-participant, male, ≥70 years, medium level of education)*
	Q A.4	*“Well, it should be somewhere close, okay? I wouldn’t want to like have to drive far to get there, that I first have to drive 20 km or so.” (non-participant, male, ≥70 years, high level of education)*
	Q A.5	*“Well, it would have to be a gym where one, we at times were, okay, one could somehow change clothes. Those who came by car were already wearing their sports clothes.” (participant, female, < 70 years, medium level of education)*
	Q A.6	*“Larger rooms. I don’t like to move around if I’m touching strangers all the time and a good room atmosphere is important to me.” (participant, female, ≥70 years, high level of education)*
	Q A.7	*“It’s very complex and time consuming.*” *(participant, female, < 70 years, medium level of education)*
	Q A.8	*“The exercises can be mastered by experienced and by untrained people of that age group. Little time is required.” (participant, male, ≥70 years, high level of education)*
	Q A.9	*“I wish it would take place once a week.*” *(non-participant, female, < 70 years, medium level of education)*
	Q A.10	*“Uh, and it has to take place at least twice a week. It can also be even three times, but I think twice a week is very important to be able to keep in tune. Plus, there is also the possibility to do the exercises at home but I am a little phlegmatic I.*”* (non-participant, female, ≥70 years, medium level of education)*
Gender-specific factors	Q G.1	*“Because it was fun to learn something about improving my physical and mental health in a group setting.” (participant, female, > 70 years, high level of education)*
	Q G.2	*“In principle, I’m a bit reserved when it comes to organized activities. I mean, groups, I am not a big group person.” (non-participant, male, ≥70 years, high level of education)*
	Q G.3	*“This is my personal …, as I said, I met two ladies who I have contact with because I had been looking for company and we will deepen this relationship.” (participant, female, > 70 years, medium level of education)*
	Q G.4	*“Not all participants have access to the internet, particularly older participants. Hard copies would have been better.*” *(participant, female, < 70 years, medium level of education)*

^a^ To improve readability the quotations have been linguistically polished to a small extent

## Results

### Characteristics of participants

The characteristics of the study population are displayed in Table 2. In total, 266 participants of the PROMOTE study completed the questionnaire assessing acceptance and 25 persons took part in the qualitative interviews in the RTC study. More than half of all the participants were female (55%) and younger than 70 years (53%). Further, 96% of the participants had a medium or high level of education. The proportion of participants meeting the PA recommendations of the WHO among PROMOTE participants was 88%. Among RTC participants, 64% reported being moderate-to-vigorous physically active four times or more every week.

## Results of the content analysis

**Fig. 1:**
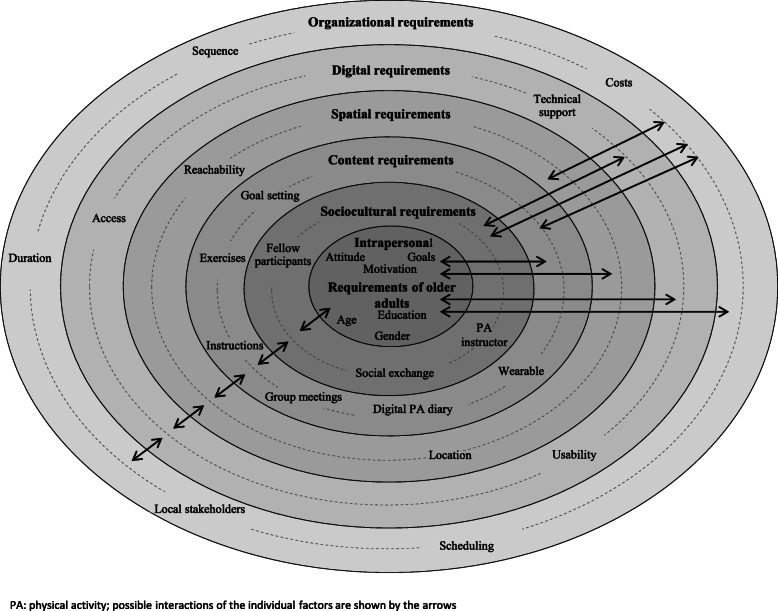
A Social-ecological model for promoting web-based physical activity interventions among older adults in Germany [own illustration based on Sallis et al. (2006) [29] and Boulton et al. (2018)] [30]

To organize all aspects raised by participants in the context of the questionnaire-based survey as well as the semi-structured interviews, a social-ecological model was created based on the work of Sallis et al. (2006) [29] and Boulton et al. (2018) [30] (Fig. 1). The model comprised six requirement levels described hereafter. For each level, the most important factors are listed with illustrative components or characteristics. The model also integrates a dynamic exchange between individuals and their environment. Possible interactions of the individual factors are shown by the arrows. Interactions can therefore occur at the same level and/or between factors at different levels. The relevant quotes from the empirical material are presented in Table 3.

### Requirements at the intrapersonal level

At this level, statements regarding attitude, motivation, and personal goals regarding participation in a web-based PA intervention were summarized. Overall, the participants had a positive view of the PA intervention. They emphasized that by participating in the intervention, health, well-being and personal body awareness were improved. In addition, they stated that participation provided the opportunity to realize personal physical deficits and get advice on how to improve one’s physical and mental fitness [Q 1.1] [Q 1.2]. Moreover, participation in the PA intervention was perceived as motivating to become more physically active in everyday life [Q 1.3]. Some participants however criticised that the PA intervention did not consider individual preferences that much, that a specific aim for PA was missing, and that the topic diet was not sufficiently addressed [Q 1.4] [Q 1.5].

### Requirements at the sociocultural level

At this level, statements about sociocultural or social requirements regarding a PA intervention were given. Respondents commented on other participants and the persons offering the intervention.

#### Other participants and social exchange

Only a few participants pointed out that their motivation to engage in regular PA was influenced by the constellation of the group, i.e. how well they related to each other. However, there was some controversy regarding the desired characteristics of fellow participants. While some preferred group meetings only with participants of the same gender, others preferred mixed groups [Q 2.1] [Q 2.2] [Q 2.3].

A further controversial point was joint participation with friends or spouses, which was liked by some respondents but disliked or even disapproved of by others [Q 2.4] [Q 2.5].

#### Trainers/exercise instructors

Regarding the trainers involved in the PA intervention, participants as well as non-participants, expected these to be persons qualified in PA. The degree of the expected qualification however varied. Several respondents expected the trainers/instructors to have certified professional qualification in the areas of medicine, PA, fitness or psychology. They stated that the level of qualification ought to be high enough to guarantee the issuing of correct instructions for the exercises as well as the correction of wrongly executed exercises. Others expected the trainers/instructors to be able to give extensive practical experiences and knowledge of various forms of PA and their effects on the body [Q 2.6]. Personal factors, such as openness, charisma, and happiness of the trainer/exercise instructor were mentioned as motivating factors [Q 2.7]. One participant felt that the trainer/exercise instructor should not play a dominant role during the intervention, but should rather offer a certain degree of openness/flexibility that differs from the usual constrictive structures of a sports club [Q 2.8].

### Requirements at the intervention content level

At this level, respondents talked about requirements regarding the content of the web-based intervention related to exercises, group meetings and specific eHealth intervention components.

#### Exercises, instructions and goal setting

Overall, the exercises chosen for the intervention, especially strength and balance exercises were deemed by the participants to be appropriate. They felt that it was advantageous that these exercises could be performed without additional props and that they could be easily integrated into everyday life [Q 3.1]. A few participants perceived the exercises as being too easy and not sufficiently challenging, while others felt that the exercises were not age-appropriate [Q 3.2].

The participants proposed various improvements that could be implemented to make the intervention more appealing. For example, they wanted better instructions for the exercises to avoid wrong execution. They also thought that short video clips demonstrating the exercises could be provided. Moreover, they wished for a stepwise entry into the PA intervention, with varying goals during the course of the intervention. The participants also suggested that a combination of physical and cognitive training and different types of PA covering muscle strengthening and coordination (e.g., Pilates) and endurance (e.g., swimming, biking) be offered [Q 3.3] [Q 3.4] [Q 3.5] [Q 3.6].

#### Group meetings

To foster positive group dynamics, participants would have liked that their fellow participants show a stronger sense of duty and a greater willingness to participate in the intervention. On the one hand some criticism was raised that the group meetings focused too much on theoretical input, on the other hand the meetings were said not to have been frequent enough [Q 3.7] [Q 3.8].

#### Specific eHealth intervention components

Participants liked the digital PA diary because it could be easily integrated into everyday life and, according to them, aided the maintenance of PA. However, some female participants pointed out that the entries in the digital PA diary were too time-consuming [Q 3.9] [Q 3.10]. Although the use of the Fitbit Zip to objectively monitor PA was regarded as motivating, there was also criticism regarding problems experienced with the installation and syncronisation of the step count program as well as the data saving [Q 3.11] [Q 3.12]. These problems were inherent in the product used.

### Requirements at the spatial level

At this level, respondents spoke about the requirements regarding physical access to the intervention. Statements were made concerning requirements regarding traveling to the intervention site, practicability and the atmosphere of the location.

#### Accessibility/reachability

Not only the distance of the intervention site from one’s home was deemed relevant, but also other factors affecting how easily (non-)participants could get there (e.g., parking, traffic) [Q 4.1]. Although respondents generally preferred an intervention near their home, some accepted longer distances, e.g., up to 20 min of travel time beyond their own area of residence. The willingness to travel longer distances increased depending on certain circumstances, such as the nice ambiance of the intervention site [Q 4.2]. While well-known and easy to reach locations were rated very positively, busy routes towards the city centre that have a lot of traffic were perceived as tiring. The degree to which distance was rated acceptable or not also depended on the availability of public transport or foot paths [Q 4.3].

#### Location

In terms of the suitability of the location for the PA program, respondents stated that the venue should have a good size, appropriate equipment as well as good ambiance (lighting, ventilation). Several participants expected the intervention to be conducted in sports facilities and were sceptical regarding the use of a general purpose building. Others were of the opinion that PA was not only possible at a sports facility, but that a closed space was sufficient [Q 4.4] [Q 4.5]. According to one participant, an optimal venue would be one that could be used during every season and could also be flexibly used for indoor and outdoor activities. It should also be located centrally so that everyday activities, such as shopping or going to the post-office, could be easily combined with participating in the intervention [Q 4.6].

### Requirements at the digital level

Participants stated that a website should be tailored to the needs of the target group and ought to be explained to participants in detail at the beginning of the intervention. They also proposed that technical supervision by trained staff should be considered, but that it should not take too much time away from the joint PA [Q 5.1] [Q 5.2] [Q 5.3].

### Requirements at the organizational level

At the organizational level, statements were made concerning the scheduling and time requirements. In addition, the requirements regarding participation of local stakeholders and potential costs for participation in the PA intervention were identified, as well as fit for the target group.

#### Sequence and duration

There was controversy among the participants regarding the general time expenditure and duration of the intervention. While some participants wanted a longer lasting intervention, others thought that the program was too long [Q 6.1] [Q 6.2].

#### Scheduling

Participants of the PROMOTE study preferred fixed appointments with a certain degree of flexibility. This need for flexibility was confirmed by responses from participants of the RTC study, who stated that they wanted to determine the scheduling for group meetings themselves [Q 6.3].

#### Local stakeholders

Above all, participants proposed the involvement of local sports clubs as an improvement of the intervention [Q 6.4].

#### Costs

Overall, participants were willing to pay for participation. For some, it did not play a role how much they would have to pay. Others however oriented themselves on the usual membership rates in a sports club. For example, 56€ per quarter or 20–30€ per month was deemed to be acceptable [Q 6.5].

### Differences in requirements by age, gender, and education

Although differences in requirements were analysed for age, gender, and education, only differences by age and gender could be identified in the statements made by the participants and non-participants.

#### Age-specific factors

Age-specific differences in requirements were detected to a similar extent at the intrapersonal, sociocultural, content, spatial and organizational levels (see Table 4). Several older adults aged 70 and above thought that a pleasant group atmosphere with mutual respect, voluntary nature, and fun was important [Q A.1]. While a number of individuals in this same age-group tended to prefer relatively homogeneous groups comprising participants of their own age, others thought it would be good to have some younger participants aged 35 to 60 years in the group [Q A.2] [Q A.3]. At the spatial level, adults over the age of 70 years preferred PA offers close to their home [Q A.4]. Among the younger respondents (< 70 years), participants felt that an intervention was doable if the size of the room was appropriate (freedom to move around during exercises without touching the next person) and the equipment was enough. This point was underlined by participants using public transport or walking to the intervention site [Q A.5]. In addition to the practicability of the intervention, the ambiance of the intervention site was also said to be important. For several individuals above the age of 70 years, the sports venue was regarded as being appropriate if the lighting, ventilation, and room size were good [Q A.6]. In terms of the time expenditure, contrasting preferences were noted. While individuals younger than 70 years thought that the intervention was too time-consuming, those belonging to the older group thought that too little time was spent participating in the intervention [Q A.7] [Q A.8]. This was also reflected in the scheduling preferences of both age groups. Individuals under the age of 70 years requested one session every week and those over the age of 70 preferred two to three sessions per week [Q A.9] [Q A.10].

**Table 4:** Overview of age- and gender-related specifics of the requirement profiles of older adults for an intervention to promote physical activity

Level ----------- Specifics	Intrapersonal requirements	Sociocultural requirements	Content requirements	Spatial requirements	Digital requirements	Organizational requirements
**≤ 70 years**	(=)	(/)	Local Group meetings (+)	Practicability of the location (+)	(=)	Time exposure (−) 1 appointment a week (+)
**> 70 years**	(=)	Pleasant group atmosphere (+); Age homogeneity (+)	(/)	Proximity of residence (+); good lighting, ventilation, and room size (+)	(=)	Time exposure (+) 2–3 appointments a week (+)
**Females**	Fun and pleasure (+)	Exchange; personal contacts (+)	Local Group meetings (+)	(=)	Digital skills (−)	(=)
**Males**	(/)	(/)	Local Group meetings (−)	(=)	(/)	(=)

(+) rather preferred, (−) rather rejected, (=) no differences identifiable, (/) no specific statement

#### Gender-related differences

There were differences between men and women regarding aspects at the intrapersonal, sociocultural, content, and digital levels (see Table 4). Female participants emphasized that fun and pleasure was an important condition for engaging in PA [Q G.1]. While men perceived PA in groups as being a barrier to engaging in PA, women regarded it as a positive factor as well as an opportunity for interaction, particularly during group meetings [Q G.2] [Q G.3]. Several female respondents were critical towards internet use and computer skills, as well as toward the time-intense use of the digital PA diary required by the intervention [Q G.4].

## Discussion

In this qualitative study, the requirements of older adults regarding web-based interventions for the promotion of PA were examined based on an extension of the social-ecological models by Sallis et al. [29] and Boulton et al. [30]. The extended social-ecological model included requirements of older adults at six different levels (intrapersonal, sociocultural, content, spatial, digital, and organizational). At the intrapersonal level, factors such as the goal to improve health, previous experiences with PA, and the motivation to increase PA during everyday life, as well as wanting to have fun while participating in the intervention were identified. These results are in line with those found in previous studies [18, 27, 30]. Offers do not always correspond with preferences, previous experiences with sports or individual performance levels. Participants of the PROMOTE study found the fact that they could not individually adapt their PA goals, intensity, or influence locations for engaging in PA difficult. This is a limitation of the PROMOTE study, considering that Rowley et al. [41] reported that the opportunity to adapt PA goals and to accompany participants throughout the program by individualizing goals led to a significant increase in PA in their study. The wish to engage socially should particularly be taken into consideration during future planning of PA interventions and made use of during advertising for the intervention. Sport is regarded as an opportunity to get in touch with the respective social environment [18]. In another study, contacts between intervention participants and the exercise instructor were observed to be of similar relevance regarding the promotion of PA in older adults [23].

Exercises without props, which could be easily integrated into everyday routines were regarded as being particularly positive. In addition to the wish for qualified staff, issues related to the need for digital content, such as video clips with instructions, were raised. Similar to other studies [23], we conclude that web-based interventions should be adapted to the needs of users and that the trainer/exercise instructor should play a mediating role [42]. Specific aspects, such as the PA diary, which was used during the PROMOTE intervention, as well as the Fitbit, were on the one hand seen as time-consuming and, on the other hand, as motivating. Similarly, results of two previous studies suggest that web-based interventions be kept as simple as possible and be implemented during a set time frame [21, 22].

In previous studies, aspects of the physical environment, such as the appeal of the location of an intervention and how easy it was to reach it [22, 24, 25], as well as the equipment used, were perceived as either barriers [22] or facilitators for PA. Similarly, the results of our study suggest that good access and certain attributes of the PA venues (appropriate size, lighting, and equipment) were relevant. At the digital level, requirements concerning access, technical support and user friendliness were raised by the participating older adults. Assuming that in the current age cohort of 65+ years computer and internet skills cannot be considered a given, an age-appropriate fit is necessary and the design of the intervention components supporting the use of the intervention (e.g., step-by-step instructions) and sufficient additional technical support [43, 44]. At the organizational level, there was great variation in the rating of the time exposure and the duration of the program. In terms of scheduling, flexible times were thought to be beneficial. In contrast to the statements in earlier research [24], some participants in this study were interested in being physically active in the evening as well. This indicates the need for individually tailored exercise time or the choice of different exercise periods as well as the design of exercise times and the choice of the venue. Our results also confirm the findings of another study [22] that economic aspects, such as costs of participation are relevant factors. Costs for the PA program participation ought to underline the relevance of the program but should not be too high for the individual person.

In addition, and also similar to the results of other studies [16, 20], a few age- and gender-specific aspects were noted in our study. Particularly the social aspect of engaging in PA in groups, such as meeting new people, appealed to women but not to men. Further, women seemed more critical about the use of the internet and computer and were less willing to spend time on this than men. Additionally, similar to another study reporting differences in preferences between online and offline meetings with fellow intervention participants [45], women in our study seemed to prefer offline meetings. Several age-specific differences emerged in our study, which were partly similar to those observed in a study by Boulton et al. [30]. These confirm the wish of younger older adults to have fewer obligations regarding appointments while transitioning from work into retirement. For older adults aged 70 years and above, the close proximity of the exercise venue to their home is very important and contributes to a sense of subjective safety (i.e., knowing the vicinity and being able to return home quickly).

### Strengths and limitations

The socio-ecological model developed for PA promotion for older adults in Germany takes various requirements for participating in a web-based PA intervention into account. One strength of this study is that the picture is enriched by the views of participants and non-participants. Furthermore, the model contains requirements at the digital level in addition to conditions of the physical environment, thereby providing recommendations which are complementary to previous socio-ecological models [29, 30] for the context of PA promotion in older adults. Due to a relatively big qualitative sample of participants and non-participants, varied results could be obtained, including age and gender differences. One limitation of the study is that a relatively high percentage of physically active, hardly ethnically diverse and well-educated individuals were included in the study (selection bias). The requirements among individuals with ethnically diverse background as well as lower levels of PA and education have yet to be determined. Another limitation is that only participants who completed the ten-week PA program in the intervention groups 1 and 2 filled out the questionnaire assessing acceptance of the intervention at follow-up. Hence, they may have had a more positive opinion of the intervention compared to those who did not complete the intervention or were participants in the waiting list control group. For the reasons outlined above, all results reported here are not generalizable to the general population of adults aged 65 years and above. Further, the non-participants statements need to be interpreted taking into account that the non-participants were more distant to the intervention (i.e., judging the content and design of the intervention based on flyers, information events in their city district and the invitation letter to the study). It is also possible that the relationship between the interviewer and the interviewee influenced some of the responses, leading to some aspects that were perceived as being negative being phrased positively. There may have also been inaccuracies while jointly analysing and interpreting the results of the survey and interview data, because some responses given in the interviews differ from answers provided in the free text field in the questionnaires.

## Conclusions for research and practice

To conclude, our findings suggest dynamic and multidirectional interactions between individual preferences of older adults at different levels, and their life circumstances. The statements of our sample contained important information on various requirement profiles, which can now be more systematically included in the process of intervention development and implementation. During the planning, implementation and sustainability phases, when choosing the location, subjective safety and accessibility, as well as the availability of parking for cars and bicycles and sanitary facilities should be taken into account. The age and gender differences in expectations expressed by our sample regarding the types and intensity of activities show that the assessment of information regarding individual preferences and everyday life circumstances before the conception of interventions is of central importance. More clarity on specific requirements could be obtained through further research specific subgroups (e.g., older adults with low levels of PA or older adults with migration background). The socio-ecological model which was developed as part of this study can serve as an orientation for stakeholders involved in the co-design of future PA interventions. Age and gender-specific needs or other characteristics of the target group could guide the selection of a varied test sample for the piloting loops of future PA interventions, as it is difficult to design universally suitable offers for older adults. Rather, targeted program specifications could be made (e.g., according to fitness level), or interventions could be developed in modular formats. The use of websites and other digital components such as activity trackers is definitely possible in this age group. However, not only different skillsets and needs for support should be considered, but also the possibility not to use these types of intervention components altogether.

## Data Availability

The datasets analysed during the current study are not publicly available because the data collection as approved by the ethic committee did not allow for making them publically available.

## Abbreviations

ACSM: American College of Sports Medicine
AEQUIPA: Physical activity and health equity: primary prevention for healthy ageing
BMBF: German Federal Ministry of Education and Research
ISCED: International Standard Classification of Education
MVPA: Moderate-to-vigorous physical activity
PA: Physical activity
RTC: Ready to Change
WHO: World Health Organization
